# The Influence of Different Caregivers on Infant Growth and Development in China

**DOI:** 10.3389/fped.2017.00243

**Published:** 2017-11-16

**Authors:** Qinrui Li, Furong Liang, Weilan Liang, Jing Zhang, Manman Niu, Ying Han

**Affiliations:** ^1^Department of Pediatrics, Peking University First Hospital, Beijing, China; ^2^Department of Child Health Care, Peking University First Hospital, Beijing, China

**Keywords:** caregivers, growth and development, infants, obesity, Denver Developmental Screening Test

## Abstract

**Objective:**

An increasing number of parents in China ask grandparents or babysitters to care for their children. Modern parents are often the only child in their family because of China’s One-Child Policy and thus may lack interaction with siblings. Accordingly, the present study aimed to explore whether different caregivers affect the physical and development of infants in China.

**Methods:**

In total, 2,514 infants were enrolled in our study. We assessed their weight-for-age, supine length-for-age, weight-for-length, occipital-frontal circumference, and Denver Developmental Screening Test (DDST) results and recorded their general parental information and their primary caregivers.

**Results:**

The weights and lengths of 12-month-old infants under the care of babysitters were significantly lower than those of infants under the care of parents or grandparents (*P* < 0.05). Additionally, 12-month-old infants under the care of babysitters had the lowest DDST pass rate (75%) among the three groups (χ^2^ = 11.819, *P* = 0.012), especially for the fine motor-adaptive and language domains. Compared to 12-month-old infants under the care of parents and babysitters, infants under the care of grandparents were more likely to be overweight or obese (*P* < 0.001).

**Conclusion:**

The study showed that caregivers had a dominant role in the physical and cognitive development of the infants. Specifically, compared with infants raised by grandparents and parents, 12-month-old infants under the care of babysitters had partially suppressed lengths and weights and lagged cognitively. The 12-month-old infants under the care of grandparents were more overweight than those cared for by parents and babysitters.

## Introduction

China’s economy has been rapidly developing in recent decades, and this development has resulted in a fast-paced and stressful living environment for many people. An increasing number of parents are busy with work and thus have little time to take care of their children. Therefore, they are turning to grandparents or babysitters to help care for their children. However, infants’ health and intellectual development may differ depending on whether their primary caregiver is a parent, grandparent, or babysitter. Inadequate psychosocial stimulation and lack of nutrients have been reported to affect child growth and development (1, 2). The first year of life lays the foundation for the future development of children, and their brains develop rapidly during this period (3). Environments surrounding children modify their brain development and influence their cognitive and emotional development (4). Furthermore, a child’s development concerns not only their family but also the future of the country. Therefore, elucidating the factors that influence infant development is urgent.

The closest people to infants are their parents, and most parents in our study were only children under China’s One-Child Policy. This policy was introduced in 1979, and for only children, has led to numerous negative outcomes, such as being more self-centered and less cooperative (5). Today, these only children are married and have infants, thus forming the “4:2:1 generation,” which refers to two married singletons who have to care for their four aging parents and one child (6). Young parents tend to provide milk to infants when they cry, even in the absence of hunger (7, 8). This emotional overeating behavior may result in negative eating behaviors and higher body mass index (9). Maternal education has been associated with child growth and development, and higher levels of maternal education have positively affected the standardized cognitive scores of children (10–12). In animal models, maternal support has been shown to promote epigenetic gene expression, neurogenesis, adaptive stress responses, methylation of the glucocorticoid receptor gene, and larger hippocampal volumes in developing animals (13–15). Boys living with their mothers had higher physical activity levels (16), and girls whose parents were divorced were more concerned about their weight than those who lived with both parents (17).

Grandparents play an important role in the family, especially under China’s One-Child Policy. Chinese grandparents treat their single grandchildren as “little emperors” and tend to over-care for them (7, 18). These grandparents express their love via food and indulge their grandchildren. Studies suggested that grandparents play a prominent role in childhood obesity by using food as an educational and emotional tool (7, 19). In the UK and America, children under the care of grandparents are also more likely to be overweight or obese (20). In certain studies, grandparents have been shown to have positive effects on the development of grandchildren. Grandparents’ involvement in their grandchildren’s learning has been associated with higher levels of social behavior and vocabularies (21).

An increasing number of parents use babysitters to care for their children while they are busy with work. Most babysitters in China have a low level of education. Additionally, babysitters are often reported to engage in mistreatment of infants because they are not relatives (e.g., in China, a babysitter was secretly filmed while kicking and hitting a 5-year-old boy and screaming “I will beat you to death” because the boy would not eat his food). Compared to parents or grandparents, babysitters interact with children less (22, 23). While it was recently established that an increasing number of babysitters in China are professionally trained, infant development has not been systemically studied.

In the present study, we explored differences in infant development and growth under the care of parents, grandparents, and babysitters. We hypothesized that infants cared for by their parents grow and develop better than those cared for by grandparents or babysitters.

## Materials and Methods

### Participants

This study was conducted at Peking University First Hospital. The data were collected from April 2014 to March 2017. The study was carried out in accordance with recommendations from the Clinical Research Ethics Committee of Peking University First Hospital, and written informed consent was acquired from all the parents and grandparents of this study. All parents provided written informed consent in accordance with the Declaration of Helsinki, and the Clinical Research Ethics Committee of Peking University First Hospital approved the protocol. The infants included in this study were full term (gestational age between 37 weeks and 42 weeks), had a normal birth weight (2,500–4,000 g), and did not have severe diseases or any abnormalities at birth. The exclusion criteria included infants with a gestational age <37 weeks, a birth weight <2,500 g, infants with diabetic mothers, a history of asphyxia at birth (Apgar < 3), or severe diseases (e.g., intracranial hemorrhage, phenylketonuria, chronic diarrhea, necrotizing enterocolitis, severe protein allergy or acute infectious diseases 2 weeks before the study). Children who met the criteria and did not meet the exclusion criteria were eligible. We collected children’s demographic characteristics, gender (male or female) and parental educational level (less than undergraduate, undergraduate, or more than undergraduate). The infants were divided into four groups according to the children’s ages: 3-month-olds, 6-month-olds, 9-month-olds, and 12-month-olds. Each group was subdivided into three subgroups according to the type of primary caregiver, who cared for the infants for more than 12 h per day for 3 months: the parent group, the grandparent group, and the babysitter group.

### Measurements of Physical Growth

The following parameters were used to evaluate growth: weight-for-age, supine length-for-age, weight-for-length, and occipital-frontal circumference (OFC). These parameters were based on the measures recommended by the WHO Multicentre Growth Reference Study Group (24). Length and weight were measured using a portable instrument (Seca, Germany), and a precision of 50 g was used to assess weight. We defined obesity as 20% above standard weight for the height of same-aged infants and overweight as 10% above standard weight for the height of same-aged infants (25, 26).

### Assessment of Ability Development

The children’s intelligence development was assessed with the Denver Developmental Screening Test (DDST). The DDST has been utilized worldwide and was standardized to the Chinese context in 1982. This scale is used to enable early identification of developmental delays in children from birth to 6 years of age. The standardized DDST consists of 104 items and covers four areas of development: (a) personal–social, (b) fine motor-adaptive, (c) language, and (d) gross motor. Children are assessed in the presence of their caregivers. In the present study, three trained professionals tested the children. The response options for the items are “passes,” “fails,” “refuses,” and “has not had the opportunity.” The results of the DDST are normal (no delays), suspect (2 or more caution items and/or 1 or more delays), abnormal (2 or more delays), or untestable (refusals of one or more items completely to the left of the age line or more than one item intersected by the age line in the 75–90% area) (30). With the exception of normal children, all other children should be retested 2 or 3 weeks later.

### Statistical Analysis

Descriptive and inferential analyses were performed using SPSS 18.0. Numerical variables are described as the mean ± SD (height, weight). Enumeration data and ranked data are described as percentages. We used analysis of variance, the χ^2^ test, and non-parametric tests to assess the differences in child development between the three groups. A value of *P* < 0.05 was considered statistically significant.

## Results

**Figure 1:**
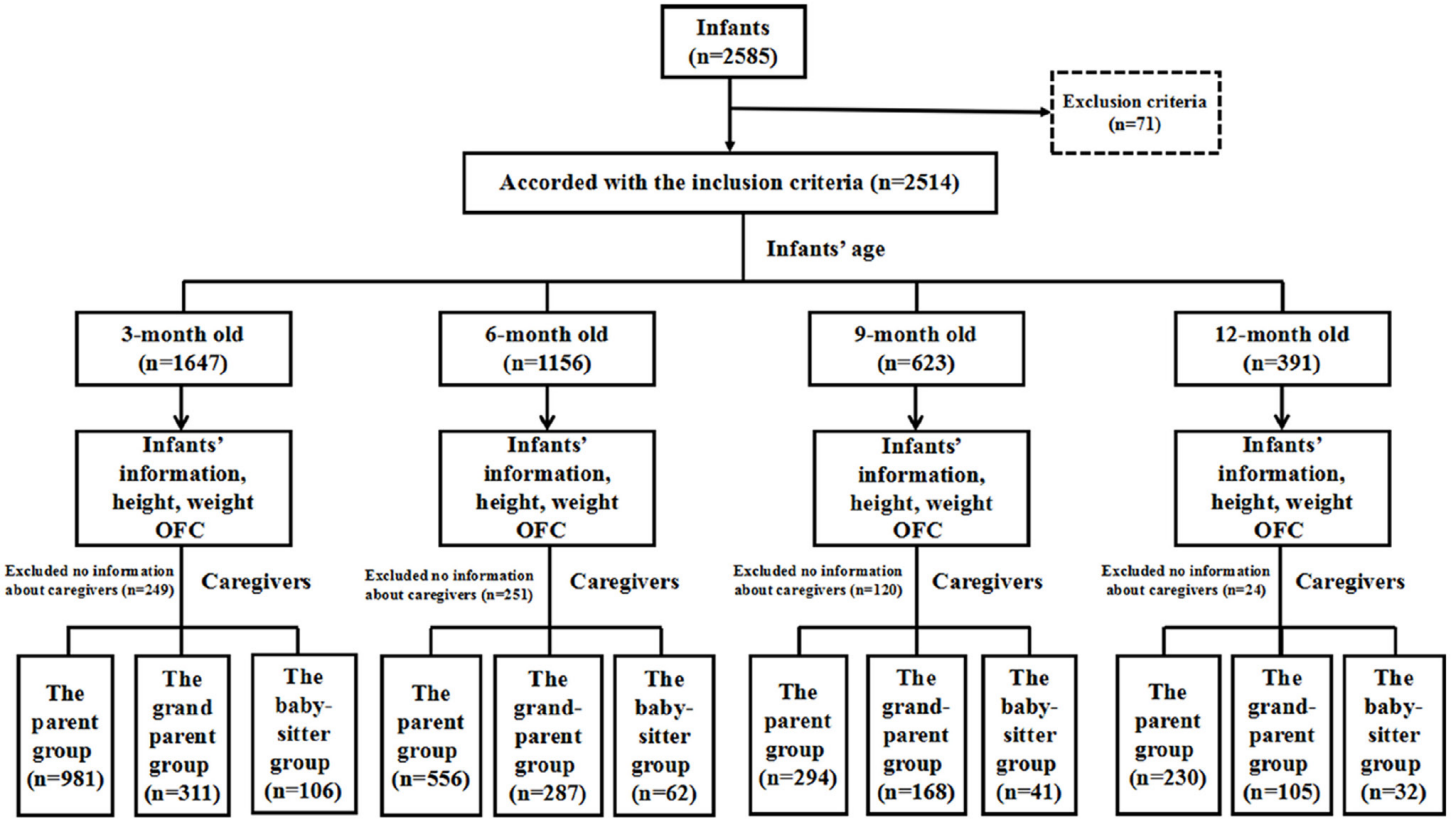
The flowchart of the study design. In total, 2,585 infants were enrolled and only 2,514 infants met the inclusion criteria. There are 1,647 infants of 3 months old, 1,156 infants of 6 months old, 623 infants of 9 months old, and 391 infants of 12 months old enrolled in our study. They were divided into three groups according to the caregiver type.

In total, 2,585 infants took part in our study and 71 infants were excluded (a gestational age <37 weeks, a birth weight <2,500 g, a history of asphyxia at birth, or severe diseases). Overall, 2,514 participants (1,328 male and 1,186 female) aged 3–12 months attending a physical health examination at Peking University First Hospital were considered eligible. Figure 1 shows the flowchart of the study design. We found the analysis and findings are on the 391 only at age 12 months. Therefore, in the study, we focused on the 391 infants aged 12 months. The average birth weight of the participants was 3.33 ± 0.37 kg, and the average birth length was 50.5 ± 1.6 cm.

In total, there were 1,647 3-month-old infants (841 male and 806 female), 1,156 (616 male and 540 female) 6-month-olds, 623 (341 male and 282 female) 9-month-olds, and 391 (191 male and 200 female infants) 12-month-olds in our study group.

Table 1 shows the influence of maternal education level and different caregivers on the weight, length and OFC of infants in the 3-, 6-, 9-, and 12-month-old groups. Infants under the care of babysitters had a significantly different 12-month weight (9.80 ± 1.75 kg) and length (76.6 ± 3.7 cm) than those under the care of parents (weight: 10.22 ± 1.09 kg and length: 77.3 ± 2.7 cm) or grandparents (weight: 10.55 ± 1.47 kg and length: 77.9 ± 2.7 cm) (*P* = 0.009 and *P* = 0.043, respectively). Head circumference did not significantly differ between the three groups (*P* = 0.212).

**Table 1:** The influence of maternal education level and different caregivers on the weight, length, and OFC of infants in the 3-, 6-, 9-, and 12-month-old group.

		3-month-old			6-month-old			9-month-old			12-month-old		
		Weight (kg)	Length (cm)	OFC (cm)	Weight (kg)	Length (cm)	OFC (cm)	Weight (kg)	Length (cm)	OFC (cm)	Weight (kg)	Length (cm)	OFC (cm)
Maternal education level	Less than undergraduate	6.82 ± 0.77	62.9 ± 2.5	40.8 ± 1.7	8.49 ± 1.08	68.7 ± 3.0	43.4 ± 1.4	9.24 ± 1.21	73.1 ± 2.9	45.0 ± 1.5	10.23 ± 1.29	77.4 ± 2.8	46.5 ± 1.4
	Undergraduate	6.80 ± 0.78	62.8 ± 2.3	40.8 ± 2.4	8.52 ± 1.00	69.0 ± 2.4	43.5 ± 1.3	9.49 ± 1.15	73.4 ± 2.8	45.3 ± 1.4	10.20 ± 1.32	77.2 ± 2.8	46.4 ± 1.4
	Higher than undergraduate	6.81 ± 0.79	62.9 ± 2.3	40.9 ± 2.5	8.42 ± 0.95	68.9 ± 2.2	43.7 ± 1.3	9.36 ± 1.04	73.2 ± 2.3	45.4 ± 1.3	10.29 ± 1.22	77.6 ± 2.8	46.4 ± 1.3
	*F*-value	0.095	0.457	0.521	0.888	1.617	2.571	2.253	0.312	2.975	0.160	0.444	0.185
	*P*-value	0.909	0.633	0.594	0.412	0.199	0.077	0.106	0.732	0.052	0.853	0.642	0.831

Caregiver group	Babysitters	6.67 ± 1.03	62.5 ± 2.4	40.8 ± 1.3	8.51 ± 1.06	68.7 ± 3.0	43.6 ± 1.4	9.19 ± 1.03	73.1 ± 2.5	45.2 ± 1.6	9.80 ± 1.75	76.6 ± 3.7	46.1 ± 1.2
	Grandparents	6.76 ± 0.84	62.9 ± 2.2	40.9 ± 1.2	8.52 ± 1.00	69.0 ± 2.4	43.5 ± 1.3	9.52 ± 1.12	73.6 ± 2.7	45.4 ± 1.3	10.55 ± 1.47	77.9 ± 2.7	46.5 ± 1.5
	Parents	6.83 ± 0.76	63.0 ± 2.4	40.8 ± 2.7	8.46 ± 0.97	69.0 ± 2.2	43.7 ± 1.3	9.47 ± 1.17	73.3 ± 2.7	45.2 ± 1.3	10.22 ± 1.09	77.3 ± 2.7	46.5 ± 1.3
	*F*-value	2.433	0.917	0.645	0.326	0.136	0.959	2.032	0.666	1.338	4.746	3.174	1.559
	*P*-value	0.088	0.400	0.525	0.722	0.872	0.384	0.132	0.514	0.263	0.009*	0.043*	0.212

*P < 0.05.

*OFC, occipital-frontal circumference*.

We analyzed the weight status of the 12-month-old infants. In total, 55 infants (14.1%) had weight problems: 39 (7%) were overweight and 16 (4.2%) were obese. The rates of overweight did not significantly differ by gender (χ^2^ = 2.031, *P* = 0.387). Table 2 describes the effect of caregiver type on infants’ weight status. Specifically, infants under the care of grandparents were more likely to develop overweight or obesity (χ^2^ = 20.576, *P* < 0.001).

**Table 2:** The influence of caregiver type on 12-month-old infants’ weight status.

Caregivers	Overweight + obesity		Obesity	
	Infants	%	Infants	%
Parents	25	0.113	3	0.014
Grandparents	29	0.264	13	0.118
Babysitters	1	0.02	0	0
χ^2^	20.576			
*P*-value	0.000*		0.000*	

*P < 0.05.

Next, we analyzed the DDST pass rates of the participants. In total, 91.5, 86.3, 84.8, and 88.2% of the children aged 3, 6, 9, and 12 months, respectively, passed the DDST. The DDST pass rates were noted in the parent, grandparent, and babysitter groups. For 3-month-old infants, the DDST pass rates were 91.9% (parent group), 91.3% (grandparent group), and 91.5% (babysitter group). The DDST pass rates in the parent, grandparent, and babysitter groups were, respectively, 89.7, 86.8, and 88.3% for 6-month-olds, and 85.4, 83.9, and 80.5 for 9-month-olds. As shown in Table 3, there was no significant difference among infants with different maternal education level. We found that 12-month-old infants under the care of babysitters had the lowest DDST pass rate (75.0%) compared with the rates in the parent (93.0%) and grandparent (86.7%) groups (χ^2^ = 11.819, *P* = 0.012). Furthermore, we investigated the pass rates of the four development areas among 12-month-old infants who had failed to pass the DDST. Table 4 shows that the 12-month-old infants in the babysitter group had lower pass rates in the fine motor-adaptive domain (75%) than infants in the parent group (91.7%) or grandparent group (86.7%) (χ^2^ = 7.929, *P* = 0.017). Additionally, the babysitter group had the lowest pass rates in the language domain (75%), with higher rates found in the parent group (90.0%) and grandparent group (76.2%) (χ^2^ = 13.136, *P* = 0.001) (Table 4).

**Table 3:** The influence of maternal education level and caregiver type on the Denver Developmental Screening Test (DDST) pass rates among the infants.

		3-month-old			6-month-old			9-month-old			12-month-old		
		Pass (*n*) (%)	Suspect (*n*) (%)	Abnormal (*n*) (%)	Pass (*n*) (%)	Suspect (*n*) (%)	Abnormal (*n*) (%)	Pass (*n*) (%)	Suspect (*n*) (%)	Abnormal (*n*) (%)	Pass (*n*) (%)	Suspect (*n*) (%)	Abnormal (*n*) (%)
Maternal education level	Less than undergraduate	253 (0.891)	17 (0.06)	14 (0.049)	128 (0.826)	12 (0.077)	15 (0.097)	87 (0.845)	10 (0.097)	6 (0.058)	56 (0.812)	10 (0.145)	3 (0.043)
	Undergraduate	787 (0.916)	42 (0.049)	30 (0.035)	578 (0.893)	35 (0.054)	34 (0.053)	312 (0.855)	25 (0.068)	28 (0.077)	174 (0.879)	14 (0.071)	10 (0.051)
	Higher than undergraduate	403 (0.929)	15 (0.035)	16 (0.037)	307 (0.885)	25 (0.072)	15 (0.043)	115 (0.816)	7 (0.005)	19 (0.135)	331 (0.886)	33 (0.079)	17 (0.035)
	χ^2^		4.092			7.876			6.791			3.859	
	*P*-value		0.392			0.093			0.142			0.421	

Caregiver type	Parents	902 (0.919)	44 (0.045)	35 (0.036)	499 (0.897)	33 (0.059)	24 (0.043)	251 (0.854)	22 (0.075)	21 (0.071)	214 (0.93)	9 (0.039)	7 (0.03)
	Grandparents	284 (0.913)	13 (0.042)	14 (0.045)	249 (0.868)	19 (0.066)	19 (0.066)	141 (0.839)	8 (0.048)	19 (0.113)	91 (0.867)	10 (0.095)	4 (0.038)
	Baby sitters	97 (0.915)	5 (0.047)	4 (0.038)	53 (0.883)	6 (0.171)	3 (0.086)	33 (0.805)	4 (0.098)	4 (0.098)	24 (0.75)	5 (0.156)	3 (0.094)
	χ^2^		0.821			2.529			4.398			11.819	
	*P*-value		0.940			0.633			0.344			0.012^a^*	

*^a^Fisher’s exact test*.

*P < 0.05.

**Table 4:** The influence of different caregiver type on the pass rates for the four Denver Developmental Screening Test (DDST) domains among 12-month-old infants.

	Gross motor (*n*) (%)	Personal–social (*n*) (%)	Language (*n*) (%)	Fine motor-adaptive (*n*) (%)
Parents	206 (0.896)	226 (0.983)	207 (0.9)	211 (0.917)
Grandparents	96 (0.914)	99 (0.943)	80 (0.762)	91 (0.867)
Babysitters	31 (0.969)	30 (0.938)	24 (0.75)	24 (0.75)
Total	333 (0.907)	355 (0.967)	311 (0.847)	326 (0.888)
χ^2^	1.506	5.063	13.136	7.929
*P*-value	0.499	0.071	0.001*	0.017*

*P < 0.05.

## Discussion

The aim of the study was to explore the effects of different caregivers on the physical and psychological development of infants in China. Our study used objective anthropometric measures and the DDST to assess children’s development. We found that 12-month-old infants under the care of babysitters had partially lower length and weight growth and lagged cognitively, and infants under the care of grandparents were more overweight than infants in the other groups. Taken together, these results show that infants raised by parents developed better than those under the care of grandparents or babysitters. The findings of this study offered insight into the relationships between the development of infants and their main caregiver and provided information to help children reach their full developmental potential.

In our study, no significant differences in obesity rates among infants less than 12 months old under the care of babysitters, parents, or grandparents were found. Such phenomena may have occurred because infants less than 1-year-old consume single foods, such as breast milk, formula milk, and supplementary infant food. However, 12-month-old infants under the care of grandparents had higher overweight and obesity rates. This can be ascribed to the fact that grandparents tend to overfeed and indulge their grandchildren. In China, the One-Child Policy and the historical deprivation experienced by people of the grandparents’ generation have resulted in the indulgence and overfeeding of their grandchildren (19). When a three-generation family has only one child, the grandparents strive to do their best for their one grandchild, and they think that fat infants are healthy because they experienced poverty and hunger in the 1960s. These grandparents prefer to provide their grandchildren’s favorite foods that are often high in calories, resulting in more obese children raised under the care of grandparents than raised by parents and babysitters (19).

Studies have indicated that children have enhanced cognitive, emotional, and social skills if parents are their primary caregivers (27). Parental involvement decreased the behavioral problems of children and improved their social skills (28). Children living with single parents are more likely to have behavioral problems (29). Abuse and neglect had been associated with physical and cognitive deficits that often last into adulthood (30, 31). In our study, we found that maternal education had no role in infants’ development, potentially because the lowest educational level of infants’ mothers was high school, which is sufficient to take care of infants. At the same time, mothers can acquire children rearing information from the internet. Furthermore, mothers with higher educational levels are probably busy with their work and have less time to take care of their infants. In future studies, we should compare the educational levels of the infant caregivers.

In the present study, we found that infants raised by babysitters had lower DDST pass rates compared with infants raised by parents and grandparents, especially regarding their fine motor-adaptive and language skills. The DDST is a developmental screener not a developmental assessment tool. Therefore, we cannot draw conclusions about development delay in these infants. Next, we should verify these results with the Gesell or Bayley scale. In 3-month-old infants, the DDST pass rates in the babysitter group were not different than those in the parent group; however, the DDST pass rates in the babysitter group had significant differences compared with those in the parent group. The differences in the DDST pass rates in the two age groups were probably due to the age of the infants. Infants’ language development was associated with lexical, morphosyntactic and pragmatic performance (32). Skills acquired do not vary in early childhood, but the pace of acquisition differs from child to child (33). Walker et al. showed that the caregiver–infant interactions facilitate early social-emotional and language development and affect whether children reach their developmental potential (34, 35). Tottenham et al. showed that children who spent more time in orphanages exhibited poor emotion regulation and increased anxiety because of their lack of interactivity with their caregivers (36). Studies have shown that children with parents that are involved in their improvements have fewer behavioral problems (28). Mother–child interactions benefited the cognitive and social-emotional development of the children (37, 38). Luby et al. found that maternal support in early childhood predicted larger hippocampal volumes among children of school age (39). Children raised by grandparents showed a higher percentage of below–normal development than those under the care of parents in Thailand (40). However, other studies have shown that grandparents directly influence their grandchildrens’ development in a positive manner. Children under the care of grandparents were found to have higher levels of cognitive, personal–social development and socioemotional development because they were talked to more frequently (41, 42). Grandparent–grandchildren interactions have also been found to improve the social skills and communication of children with autism spectrum disorder (43). Therefore, regardless of who provides care, caregivers should interact often with infants.

In summary, caregivers should provide an environment that supports infants’ cognitive and social-emotional development; this type of environment can help infants reach their developmental potential. First, the caregivers should provide the infants enough nutrition. Next, effective interactions with infants will improve the development of the next generation.

Our study also had certain limitations. The DDST is a developmental screener, and it is not a developmental assessment tool. Therefore, we only used the DDST to screen the development of infants. Next, we should use developmental assessment (e.g., the Gesell scale or Bayley scale). The sample in our study was small, with only 32 in the babysitter group at 12 months. Our study (3-, 6-, 9-, and 12-month-olds) was cross-sectional. Several infants continuously took part in our study at 3-, 6-, 9-, and 12-month-olds. Of the 2,514 infants, most participated in our study once or twice. To avoid the sample being too small, we used a cross-sectional design and observed the development of infants aged at 12 months. Furthermore, Chinese parents thought the babysitters would abuse their infants because many news articles about babysitters mistreating infants have been reported online (http://www.dailymail.co.uk/news/article-2885024/Babysitter-secretly-filmed-kicking-hitting-five-year-old-boy-screaming-beat-death.html). Therefore, the parents do not prefer to employ babysitters. In the future, we should enroll more participants to avoid sample bias. Specifically, people in China have different nationalities, which vary by geographic location, and have various dietary traditions, but our study was limited to the situation in Beijing. Further studies should focus on low-income and middle-income provinces in China.

## Ethics Statement

The study was carried out in accordance with recommendations from the Clinical Research Ethics Committee of Peking University First Hospital, and written informed consent was acquired from all the parents and grandparents of this study. All parents provided written informed consent in accordance with the Declaration of Helsinki, and the Clinical Research Ethics Committee of Peking University First Hospital approved the protocol.

## Author Contributions

QL conducted the experiments, analyzed the data, wrote the manuscript, and made the final approval of the version to be published. YH contributed to the conception and design of the experiment, acquired the data, revised the manuscript, made the final approval of the version to be published, and agreed to be accountable for all aspects of the work. FL contributed to the conception and design of the experiment, acquired the data, critically revised the manuscript, and made the final approval of the version to be published. WL contributed to the conception and design of the experiment, acquired the data, critically revised the manuscript, and made the final approval of the version to be published. JZ and MN contributed to the conception and design of the experiment and made the final approval of the version to be published.

## Conflict of Interest Statement

The authors declare that the research was conducted in the absence of any commercial or financial relationships that could be construed as a potential conflict of interest.

## Abbreviations

OFC, occipital-frontal circumference; DDST, Denver Developmental Screening Test; ANOVA, analysis of variance.
